# Retinoic acid improves baseline barrier function and attenuates TNF-α-induced barrier leak in human bronchial epithelial cell culture model, 16HBE 14o-

**DOI:** 10.1371/journal.pone.0242536

**Published:** 2020-12-10

**Authors:** Patrick J. Callaghan, Elizabeth Rybakovsky, Bryan Ferrick, Sunil Thomas, James M. Mullin

**Affiliations:** 1 Lankenau Institute for Medical Research, Wynnewood, PA, United States of America; 2 Department of Biomedical Engineering, Drexel University, Philadelphia, PA, United States of America; Emory University School of Medicine, UNITED STATES

## Abstract

Retinoic acid (RA) has been shown to improve epithelial and endothelial barrier function and development and even suppress damage inflicted by inflammation on these barriers through regulating immune cell activity. This paper thus sought to determine whether RA could improve baseline barrier function and attenuate TNF-α-induced barrier leak in the human bronchial epithelial cell culture model, 16HBE14o- (16HBE). We show for the first time that RA increases baseline barrier function of these cell layers indicated by an 89% increase in transepithelial electrical resistance (TER) and 22% decrease in ^14^C-mannitol flux. A simultaneous, RA-induced 70% increase in claudin-4 attests to RA affecting the tight junctional (TJ) complex itself. RA was also effective in alleviating TNF-α-induced 16HBE barrier leak, attenuating 60% of the TNF-α-induced leak to ^14^C-mannitol and 80% of the leak to ^14^C-inulin. Interleukin-6-induced barrier leak was also reduced by RA. Treatment of 16HBE cell layers with TNF-α resulted in dramatic decrease in immunostaining for occludin and claudin-4, as well as a downward “band-shift” in occludin Western immunoblots. The presence of RA partially reversed TNF-α’s effects on these select TJ proteins. Lastly, RA completely abrogated the TNF-α-induced increase in ERK-1,2 phosphorylation without significantly decreasing the TNF-driven increase in total ERK-1,2. This study suggests RA could be effective as a prophylactic agent in minimizing airway barrier leak and as a therapeutic in preventing leak triggered by inflammatory cascades. Given the growing literature suggesting a “cytokine storm” may be related to COVID-19 morbidity, RA may be a useful adjuvant for use with anti-viral therapies.

## Introduction

Epithelial tissues are physical barriers between the outside environment and our systemic circulation and interstitial tissue. Examples are the epidermis of the skin and the linings of organs defining luminal spaces in our body such as our gastrointestinal tract, renal tubes, and alveoli in the lungs [1]. Cells constituting these protective sheets are unique in that they polarize (form a distinct apical side directed toward the lumen and a basolateral side connected to an underlying basement membrane) and form specialized junctional seals termed tight junctions (TJ) around and between adjacent cells [2]. A family of proteins including tricellulin, occludin, and 27 distinct claudins comprise the barrier element of TJs in a variety of different combinations throughout different epithelia [1–3]. These proteins extend from the cell into the intercellular space, forming oligomeric complexes with TJ proteins of adjacent epithelial cells to create a circumferential seal around the subapical perimeter [1–5]. Establishment of apico-basal polarity and regulation of paracellular permeability to fluid and solutes (barrier function) is dependent on these TJ proteins and their intracellular complexes [1–5]. TJs are pivotal in the development and maintenance of epithelial barriers throughout the body, and epithelial barriers are vital to any organism’s homeostasis. Conversely, epithelial barrier leak is associated with disease in many different organ systems such as inflammatory bowel disease in the gastrointestinal tract, acute renal failure in the kidneys, and acute respiratory distress syndrome (ARDS) in the lungs [2, 6].

The airway epithelium limits infiltration of inhaled noxious stimuli into underlying tissue and vasculature, and in the process prevents damaging immune reactions [1, 7]. In addition to inhibiting pathogens, allergens, carcinogens, and other detrimental compounds from entering the body, this barrier also prevents interstitial and vascular fluid from leaking into the lungs and interfering with gas exchange [4, 5]. Altered structure/function of this barrier and its associated TJs is observed in a variety of lung disorders such as rhinitis, chronic obstructive pulmonary disease (COPD), allergic asthma, ARDS, and lung cancer [4, 7, 8–10]. The life-threatening nature of these diseases highlights why it is crucial to maintain an intact epithelium that lines the airways and alveoli and restore any compromised barrier function.

16HBE14o- (16HBE) is an immortalized human bronchial epithelial cell line obtained from a 1-year old male and has served as a longstanding model for the study of various lung disorders, a number of which relate to epithelial barrier function [11–16].

Retinoic acid (RA), a biologically active form of vitamin A, has been shown to promote the development and/or elevate the strength of a variety of epithelial and endothelial barriers [17–20]. While RA has been shown to be effective in reducing inflammation in the airway, these studies have mostly focused on it modulating immune cell differentiation/activity rather than on epithelial cells and barrier function per se [21–23]. This study sought to test whether this micronutrient could be useful in elevating basal barrier strength of this bronchial cell culture model as well as determine whether RA can alleviate 16HBE barrier leak triggered by proinflammatory cytokines such as TNF-α and Interleukin-6 (IL-6).

## Methods and materials

### Cell culture

The 16HBE cell culture was obtained from Millipore Sigma (St. Louis, MO). Cells were passaged weekly for no more than 17 weeks before returning to frozen cell stocks. This correlates to actual serial passages 47–64 from the establishment of the original immortalized cell line. No discernible changes in morphology or physiology were observed within this window of passages. After reaching confluence, cells were trypsinized (0.25% trypsin and 2.2mM EDTA) (Corning Cellgro, Manassas, VA) and then passaged on a weekly basis by seeding 3.0 x 106 cells per Falcon 150 cm2 culture flask with 50ml of Dulbecco’s Modified Minimum Essential Medium (Corning Cellgro, Manassas, VA), supplemented with 2mM L-Glutamine (Corning Cellgro, Manassas, VA), 10% fetal bovine serum (Seradigm, VMR, Inc., Radnor, PA), 1% non-essential amino acids (Corning Cellgro, Manassas, VA), and 1mM sodium pyruvate (Corning Cellgro, Manassas, VA). Cultures were incubated at 37°C in 95% air/5% CO_2_ atmosphere. Confluent cell density was approximately 3.3x10^5^ cells/cm^2^.

### Transepithelial permeability measurements

Cells were seeded for transepithelial electrical resistance (TER) and radiolabeled flux measurements into sterile Millicell polycarbonate (PCF) cell culture inserts (30mm diameter with 0.4 μm pore size) [EMD Millipore, Burlington, MA] on day 0 at a seeding density of 2.0 x 106 cells/insert. Four Millicell PCF inserts were placed in a 100mm petri dish. On day 1, all cell layers were refed with medium containing 50U/ml penicillin and 50μg/ml streptomycin (2ml apical, 15ml basolateral). The same refeed procedure was performed on day 4. All treatments with TNF-α or RA were begun on day 5 or 6 (when the cell layers were post-confluent).

Cell layers were refed with control medium on the morning of experiments and allowed to incubate at 37°C for 90-minutes prior to electrophysiological readings. Transepithelial potential difference was measured at 37°C using 1M NaCl salt bridges in series with calomel electrodes. TER was measured at room temperature (RT) using 1 second, 40 μamp, direct current pulses (through 1M NaCl salt bridges in series with Ag/AgCl electrodes) in a custom-made polycarbonate chamber designed to hold the Millicell PCF inserts. With current-passing and voltage-measuring salt bridges positioned above and below the center point of the cell layers, voltage deflections and Ohm’s law were used to calculate TER (V = iR).

Following TER measurements, the basal-lateral medium was removed and replaced with 15ml of medium containing 0.1mM, 0.1μCi/ml 14C-D-mannitol (Perkin-Elmer, Boston, MA), 0.1mM, 0.1μCi/ml 3H-lactulose (D-galactose) (American Radiolabeled Chemicals, Inc., St. Louis, MO), or 0.1mM, 0.1 uCi/ml ^14^C-inulin (Perkin-Elmer, Boston, MA) and incubated at 37°C. Triplicate 50μl samples were taken from the basolateral medium to determine the specific activity via liquid scintillation counting (LSC). Duplicate 250μl samples were taken from the apical medium at either 90 or 180 minutes for liquid scintillation counting (LSC) to determine transepithelial flux rates (J_m_). The flux rate (in picomoles/min/cm2) was calculated for the aforementioned probes diffusing across the cell layer.

### Treatment with TNF-α

Medium containing the cytokines, TNF-α and IL-6 (Peprotech, Inc., Rocky Hill, NJ), at a concentration of 125ng/ml and 200 ng/ml, respectively, were applied to the apical and basolateral cell surfaces day 6 post-seeding. Physiological measurements were taken at 24-, and 48- hours after the initial exposure. For experiments observing pERK expression, cell layers exposed to TNF-α were harvested at 30 minutes, 60 minutes, and 4 hours after initial exposure. Both TNF-α and IL-6 were dissolved directly in culture medium.

### Treatment with retinoic acid

All-trans RA (EMD Millipore, Burlington, MA) was dissolved in 100% EtOH to make a 33.3mM stock and then diluted to concentrations of 5-, 15-, or 50uM in culture medium. The appropriate concentration was then added to the apical and basolateral compartments. Physiological measurements were taken at 24- and 48-hours after initial exposure. In all experiments using RA, the controls reported are actually solvent (ethanol-containing) controls. The ethanol concentrations were made equal in all conditions, and never exceeded 0.5% (v/v).

### TNF-α enzyme linked immunosorbent assay

Basal-lateral culture medium samples from control or RA-treated cell layers were assessed for TNF-α concentration by ELISA (Invitrogen, part of Thermo Fisher). 50uL of biotinylated antibody reagent were added to each well of the kit, then 50uL of supernatant or TNF-α standard were added to each well. After incubating at room temperature for 2-hours, each well was rinsed with wash buffer. After three washes, 100uL of 0.25% strepdavin-HRP was added to each well for 30-minutes. After rinsing with wash buffer three times, 100uL of TMB substrate was added to each well and the plate was placed in the dark for 30-minutes. Finally, 100uL of stop solution was added to each well and the plate was read at 450nm using a plate reader.

### Immunofluorescent staining of occludin and claudin-4

Cells were seeded at 2.0e^5^ cells per 2.55 cm^2^ glass cover slip (Fisher Scientific, Waltham, MA). Cell layers were washed 3X in PBS. 2mL of 4% formalin were added to cover each cell layer for 3-minutes. The cell layer was once again washed three times with PBS and then blocked with 1% goat serum (Jackson ImmunoResearch, West Grove, PA). After blocking, preparations were exposed to rabbit anti-human occludin (1:300 dilution in 1% goat serum) and mouse anti-human claudin-4 (1:300 dilution in 1% goat serum) for 40-minutes *(*Fisher Scientific, Waltham, MA), washed and exposed to Alexa-flour 488 (green) goat anti-rabbit (1:400 dilution in 1% goat serum) and Cyanine3 (red) goat anti-mouse (1:400 dilution in 1% goat serum) for 40-minutes (Fisher Scientific, Waltham, MA). Finally, cell layers were exposed to DAPI (Fisher Scientific, Waltham, MA) for 1-minute, washed three times in PBS, and stored overnight at 4°C.

The following day, green and red fluorescence were observed via confocal laser scanning microscopy (Nikon A1 HD25 confocal microscope Melville, NY) and images were obtained using Nikon NIS Elements Viewer.

### Immunoblot analysis of claudin-4, occludin, ERK-1,2 and phosphorylated ERK-1,2

For occludin and claudin-4 analyses, cells were harvested from Millicell PCF inserts by washing 5X in cold PBS. Then 500μL of Buffer A with protease and phosphatase inhibitors were added to each PCF [24]. The cell layer was physically scraped away from the filter and the suspension was collected, sonicated, and ultracentrifuged. The supernatant was transferred to a separate tube and prepared for analysis by PAGE (cytosolic fraction). 300μl of lysis buffer was then added to the pellet, which was then placed on a rotator for 90 minutes and ultracentrifuged. The supernatant (detergent-soluble fraction) was removed to a separate tube and prepared for analysis by PAGE.

For ERK-1,2 and p-ERK1,2 analyses, cells were harvested from Millicell PCF inserts by washing 5X in cold PBS. Then 500μL of lysis buffer with protease and phosphatase inhibitors were added to each PCF. The cell layer was physically scraped away from the filter, and the suspension was collected, flash-frozen, and stored at −80°C. Whole-cell lysates were prepared by sonication and ultracentrifugation.

Samples of all of these lysates were analyzed by PAGE using a 4−20% gradient Tris-glycine gel (Invitrogen, a division of Thermo Fisher Scientific) at 120 V for 1 h 20 min. Precision Plus Kaleidoscope Protein Standards (Bio-Rad, Inc., Hercules, CA) were also included on each gel. Proteins were transferred at 30 V for 1 h from the gel to a nitrocellulose membrane. The membranes were then washed three times with PBS-T (0.3% Tween 20) for 10 min each and blocked with 5% milk/PBS-T at RT for 1 hr. Membranes were incubated with the specific primary antibody (occludin, claudin-4 and phospho-ERK1,2, from Fisher Scientific) at 0.5 μg/mL in 5% milk/PBS-T for overnight incubation at 4°C. (Anti-ERK-1,2 in 5% bovine serum albumin/PBST was a product of Cell Signaling, Inc.). The membranes were again washed 3X, for 10 minutes each with PBS-T, and then incubated with the secondary antibody (rabbit anti-mouse- or goat anti-rabbit-IgG antibody labeled with horseradish peroxidase, from Southern Biotech, Birmingham, AL) for 1 h at RT. The membranes were washed four times, 10 min each with PBS-T, and then treated for 1 min with Western Lightning Plus-ECL chemiluminescence reagents (PerkinElmer). The membranes and band densities were quantified on the BioRad ChemiDoc Imaging System. Band densities of the normalized experimentally-treated cell samples were compared to normalized averages of the corresponding control cell sample densities.

In place of a traditional loading control such as GAPDH or actin, we routinely use densitometry of MemCode (Thermo Fisher, Inc.) staining of total protein on our immunoblots as described by [25]. Optical density of protein bands such as occludin or pERK are normalized based upon total protein staining (bands) of the immunoblot in question, quantitating the optical density of (total) protein bands in molecular weight ranges close to the proteins of interest.

### Statistics

All reported statistics utilized paired Student’s t-tests (experimental condition vs appropriate control), with all data expressed as the mean ± standard error of the mean. N/S (not significant): P > 0.05.

## Results

### Retinoic acid enhances 16HBE barrier strength and suppresses endogenous TNF-α production

**Fig 1:**
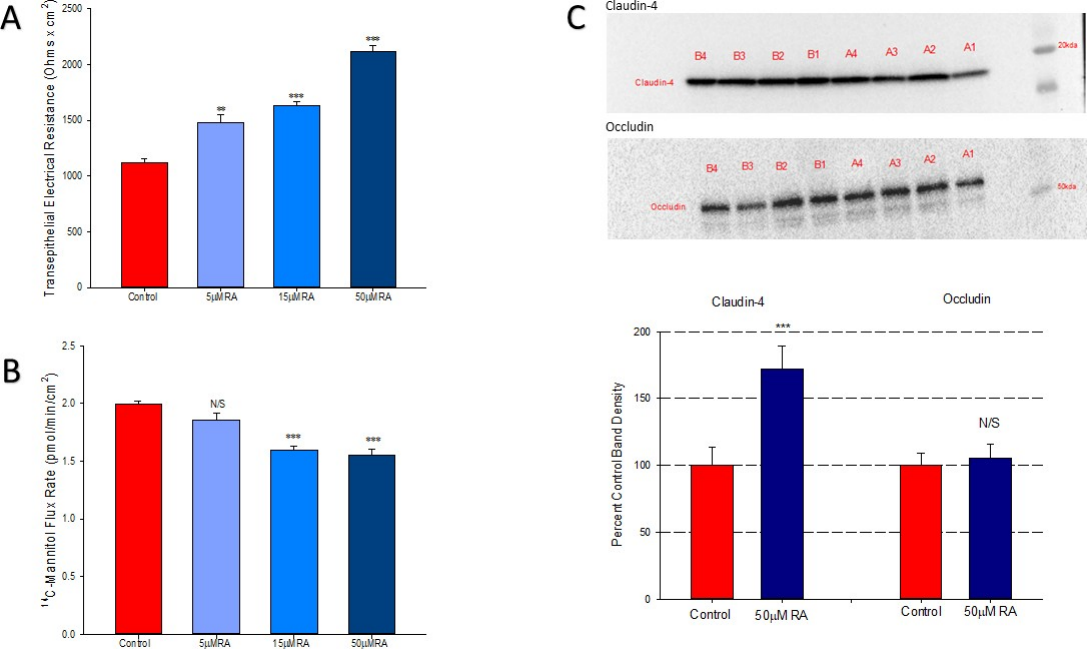
Effect of retinoic acid on 16HBE barrier function. Transepithelial electrical resistance (A) and flux of ^14^C-mannitol (B) were measured as described in Materials and Methods 48 hours after treatment. *n* = 4 cell layers per condition. Western immunoblots (C) were performed for the tight junctional proteins, occludin and claudin-4, on total cell lysates from cell layers treated for 48 hrs with 50 μM RA, n = 4 cell layers per condition. Bars indicate the mean ± standard error of the mean. N/S indicates no significant difference between experimental and control condition, ** indicates P<0.01, *** indicates P<0.001 (Student’s t test, two-tailed). RA: retinoic acid.

**Fig 2:**
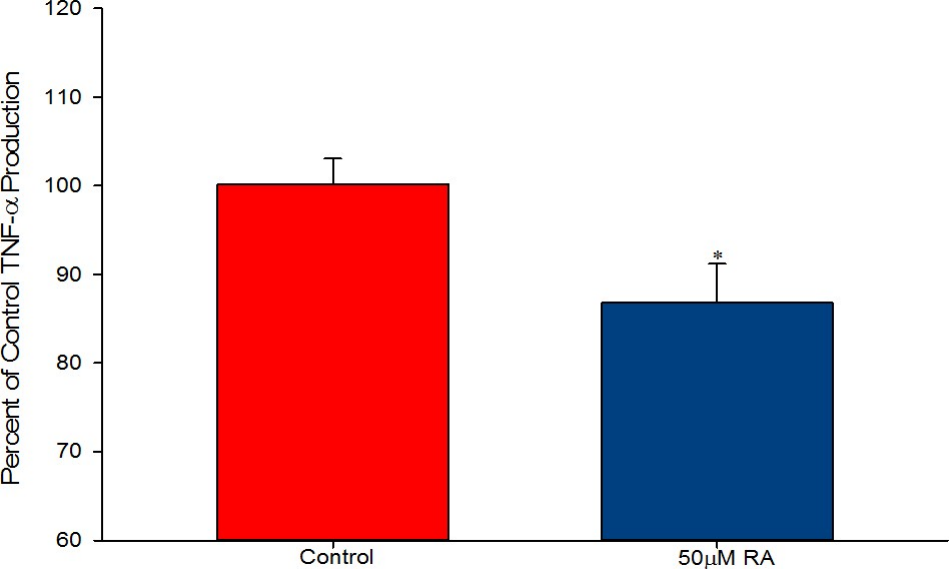
Effect of retinoic acid on endogenous 16HBE TNF-α production. TNF-α levels were measured as described in Materials and Methods 24 hours after treatment. Data are expressed as percent of mean control value. * indicates P<0.05 (Student’s t test, two-tailed). RA: retinoic acid.

Retinoic acid improved barrier strength of 16HBE in a dose-dependent manner 48-hours after exposure. At a concentration of 50μM, RA increased TER by 89% (Fig 1A) and reduced mannitol flux by 22% (Fig 1B), indicating a true increase in barrier function. Simultaneously, RA significantly increased levels of claudin-4 (CL-4), though without any effect on occludin (Fig 1C). While no concentration of RA significantly influenced barrier function at 24 hours (S1 File), RA was found to significantly reduce endogenous TNF-α production by 13% at this timepoint (Fig 2).

### Retinoic acid alleviates TNF-α-induced 16HBE barrier leak

**Fig 3:**
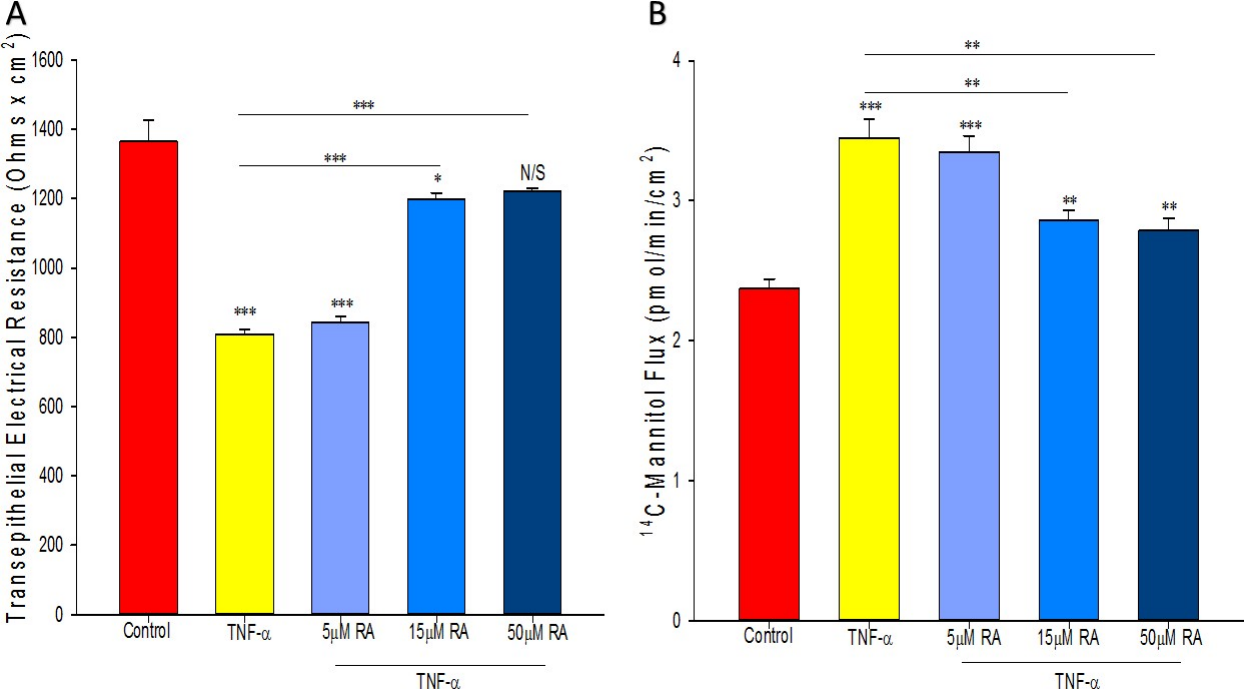
Effect of retinoic acid on TNF-α-induced 16HBE barrier leak. TER (A) and transepithelial flux of ^14^C-mannitol (B) were measured as described in Materials and Methods 48 hours after treatment. *n* = 4 cell layers per condition, N/S indicates no significant difference between experimental and control condition, * indicates P<0.05, ** indicates P<0.01, *** indicates P<0.001 (Student’s t test, two-tailed). RA: retinoic acid.

TNF-α consistently produced leak in 16HBE at both 24 and 48 hours after exposure. Like its time course of action on baseline barrier strength, RA did not significantly alter TNF-α’s effect on barrier function until 48 hours. At 48 hours, RA alleviated both the decline in TER and the increase in mannitol flux induced by TNF-α in a dose-dependent manner, with 50μM RA attenuating the reduction in TER and the increase in mannitol by 74% and 60% respectively (Fig 3).

**Fig 4:**
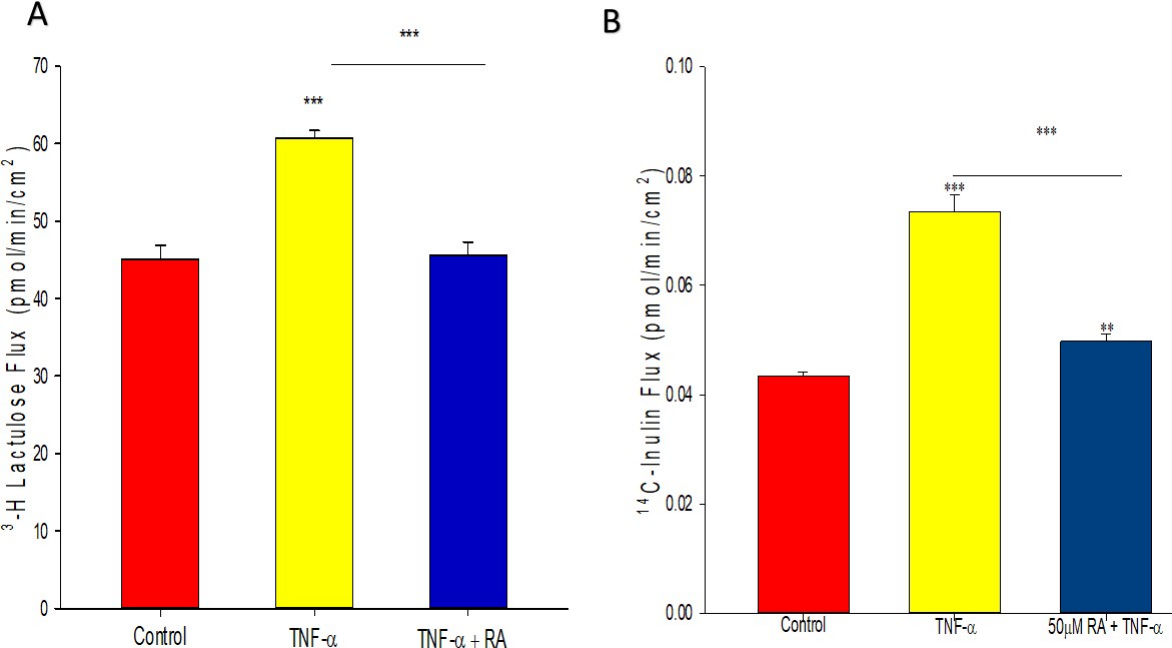
Effect of TNF-α and retinoic acid on flux of ^3^H-lactulose and ^14^C-inulin across 16HBE cell layers. Transepithelial flux of ^3^H-lactulose (A) and ^14^C-inulin (B) were measured as described in Materials and Methods, 48 hours after TNF exposure. Simultaneous incubation with 50 μM RA inhibited the TNF-induced permeability increase to both probe molecules. *n* = 4 cell layers per condition, *** indicates P<0.001 (Student’s t test, two-tailed). RA: retinoic acid.

In addition to having increased permeability to mannitol (mw 182), 16HBE cell layers treated with TNF-α were leakier to larger molecular weight probes such as lactulose (mw 342) and inulin (mw 5,500) (Fig 4). At 48 hours, lactulose flux was increased by approximately 35% after TNF exposure while inulin flux was increased over 50%. As was true for D-mannitol, retinoic acid almost completely inhibited the TNF-induced increases in both lactulose and inulin flux (Fig 4A and 4B).

### Retinoic acid inhibits the TNF-α-induced decreased immunostaining of occludin and claudin-4

**Fig 5:**
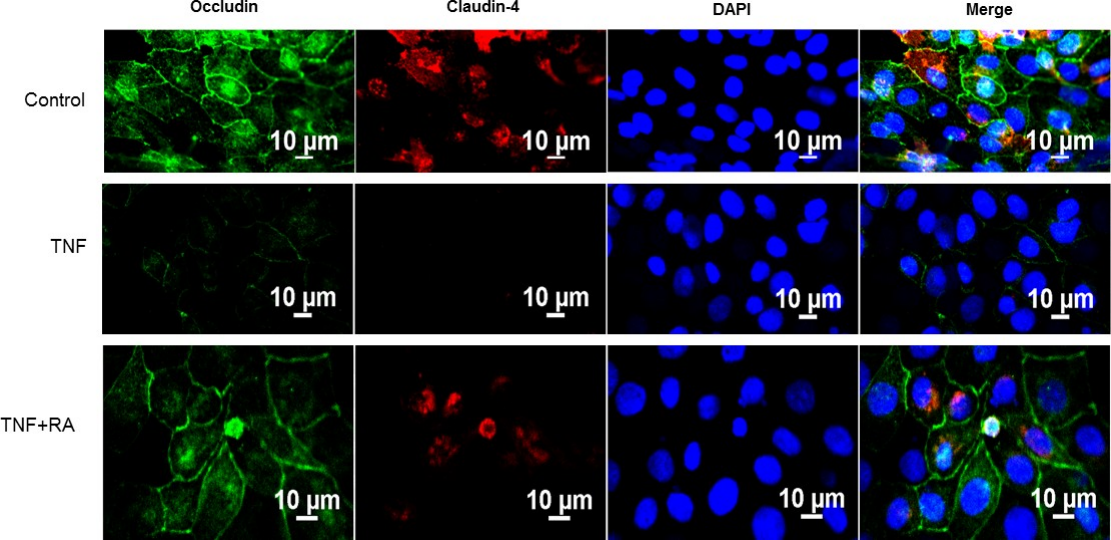
Effect of retinoic acid on TNF-α-induced 16HBE TJ disruption. Cell layers were treated with TNF-α or TNF-α and RA as described in Materials and Methods after reaching confluence. After 24 hours, staining for occludin (green), claudin-4 (red), and DAPI (blue) were performed and images observed as described in Materials and Methods. RA: 50μM retinoic acid.

In Calu-3 (immortalized bronchial cell culture model), primary bronchial epithelial cells, and intestinal HT-29/B6 cells, changes in transepithelial permeability caused by TNF-α have been shown to correlate with changes in TJ structure [26–28]. Here, it was tested whether similar changes would be observed in 16HBE and whether RA could exert any influence on TJ structure in the presence of TNF-α. Immunofluorescence methods applied to control cell layers revealed well-formed perijunctional staining of occludin, while staining for claudin-4 showed higher immunoreactivity within the cell (Fig 5). Cell layers treated with TNF-α for 24 hours were observed to have severely diminished intensity for both occludin and claudin-4. The addition of RA appeared to blunt both these effects of TNF-α, preserving immunostaining of both of these TJ proteins as well as occludin’s perijunctional ring localization specifically.

### Retinoic acid reverses the downward band-shift of occludin triggered by TNF-α

**Fig 6:**
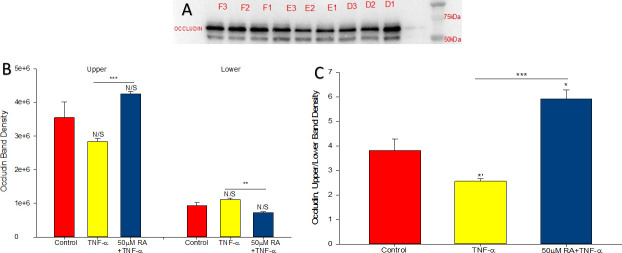
Effect of TNF-α or TNF-α and retinoic acid on occludin band-shift in detergent soluble 16HBE cell fractions. Confluent cell layers were treated for 48 hours with TNF-α or TNF-α + RA as described in Materials and Methods. Western immunoblots for occludin in detergent soluble fractions of cell layers were produced as described in Materials and Methods. (A. Control cell samples [lanes D1-D3] TNF-α-treated cell samples [lanes E1-E3], and TNF-α + RA-treated cell samples [lanes F1-F3]; B. band densities of upper and lower bands were obtained; C. the ratios of upper to lower band densities). *n* = 3 cell layers per condition; N/S indicates no significant difference between experimental and control conditions; *** indicates P < 0.001 (Student’s t test, two-tailed); *’ indicates P < 0.05 (Student’s t test, one-tailed).

TNF-α has been shown to produce a “band-shift” for occludin in Western immunoblots, namely a change in the band densities of two occludin phosphoproteins. This suggests TNF-α may play a role in altering the phosphorylation state for this TJ protein [29]. Occludin band densities were analyzed in detergent soluble fractions of 16HBE cell layers to determine whether any changes in intensity would be observed in occludin protein bands present in TJs. As shown in Fig 6A, two prominent bands were observed in Western immunoblots of occludin in detergent soluble fractions. Prior work by our group and others suggests that these bands represent phosphoproteins of occludin [30]. After 48 hours of exposure, TNF-α induced an obvious decrease in the upper band intensity while slightly increasing the lower band density (downward band-shift) (Fig 6). The proportion of upper to lower band densities were quantified, which revealed that TNF-α produced a 33% decrease in the ratio while the addition of RA produced a 55% increase, indicating that TNF-α and the combination of TNF-α and RA are modulating occludin phosphorylation in opposite directions (Fig 6C).

### Retinoic acid blocks the TNF-α-induced increase in phosphorylated ERK-1,2 expression

**Fig 7:**
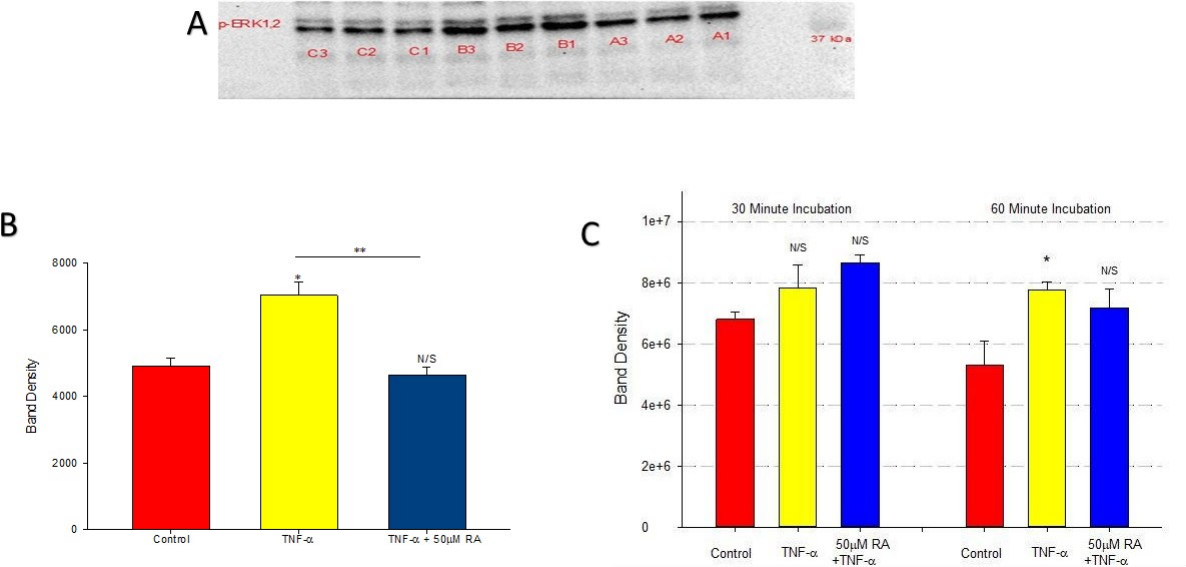
Effect of TNF-α or TNF-α + retinoic acid on phosphorylated ERK-1,2 expression in 16HBE cell layers. Confluent cell layers were treated with control or 50μM RA-supplemented medium for 24-hours prior to treatment with TNF-α or TNF-α + RA for 30 minutes. Phosphorylated ERK-1,2 immunoblots were prepared (A) and band densities were quantified (B) as described in Materials and Methods. (Lanes A1-A3 represents control, lanes B1-B3 represents TNF-α, and lanes C1-C3 represents pre-treatment with RA prior to TNF-α). Panel C shows the effects of the TNF ± RA exposure on total ERK-1,2 levels, evidencing a relatively rapid, TNF-induced, 50% increase in total ERK at 60 minutes without a significant reduction of total ERK-1,2 by RA. * indicates P < 0.05, **indicates P < 0.01 (Student’s t test, two-tailed).

Activation of extracellular-signal-regulated kinase (ERK-1,2) has been implicated in cytokine-induced TJ disassembly in Calu-3 and other epithelial cell culture models [28]. Here in 16HBE, TNF-α increased the amount of phosphorylated ERK-1,2 by approximately 40% at 30-minutes (Fig 7).This effect was completely abrogated by 24 hour pre-treatment with RA (Fig 7).This effect by TNF-α was constant through one hour, but then completely dissipated by four hours (S1 and S2 Figs).

Examining levels of total ERK-1,2 shows that TNF is likewise inducing an increase in total ERK-1,2 levels by 60 mins of TNF exposure (Fig 7, Panel C) as well as increasing pERK levels. However, 50 μM RA did not significantly reduce the total ERK-1,2 levels increased by TNF exposure, unlike the case with pERK (Panels A & B), suggesting that RA is in fact blocking the increased ERK-1,2 phosphorylation seen with TNF.

### Effect of interleukin-6 on 16HBE barrier function

**Fig 8:**
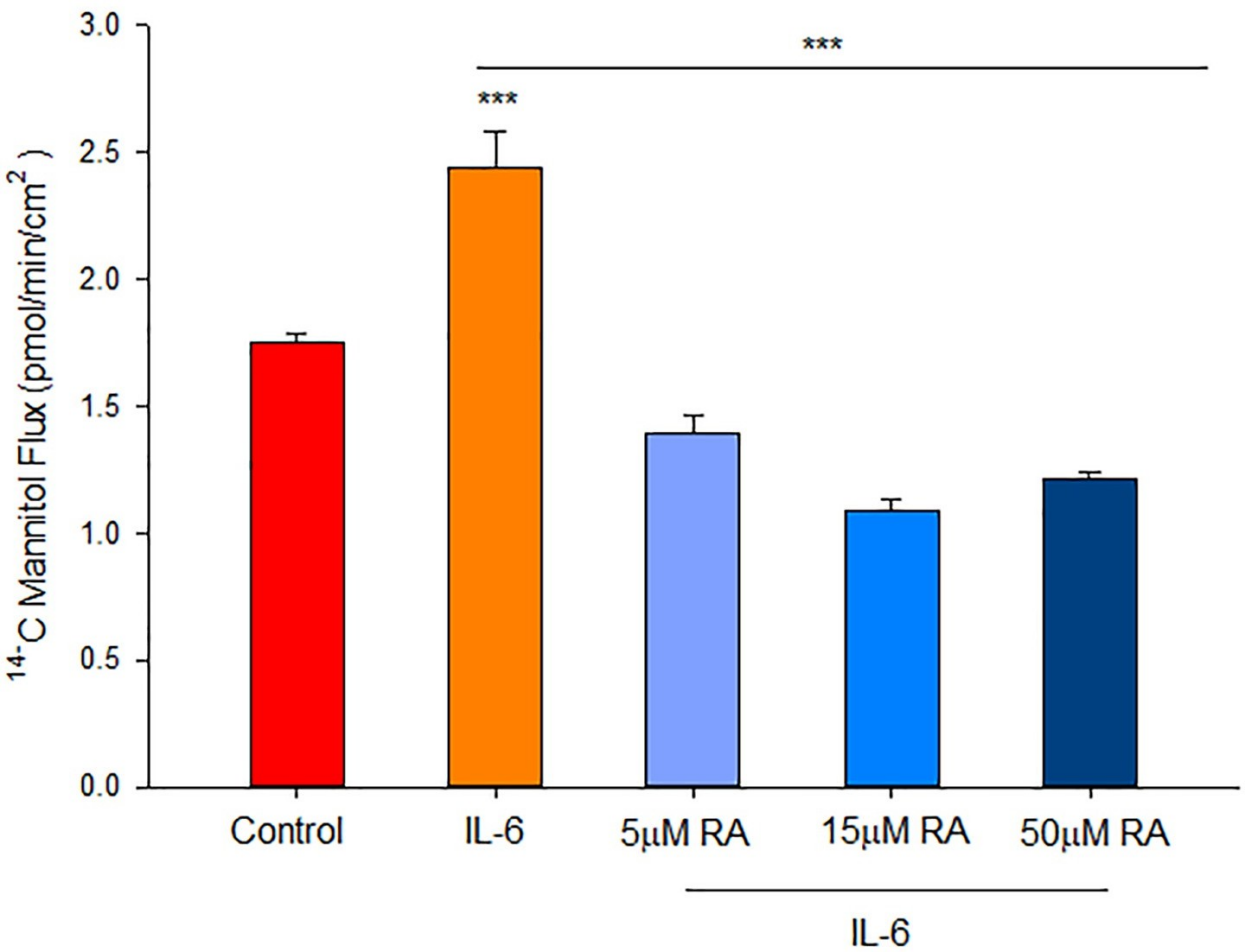
Effect of retinoic acid on IL-6-induced 16HBE barrier leak. Transepithelial flux of ^14^C-mannitol was measured as described in Materials and Methods, 48-hours after treatment. n = 4 cell layers per condition, ***indicates P<0.001 (Student’s test, two-tailed) for IL-6 vs. IL-6 + RA (all concentrations).

The strong association of elevated systemic IL-6 levels with COVID-19 infection [31, 32] prompted an examination of IL-6 effects on 16HBE barrier function as well as possible antagonism of that induced leakiness by RA. As shown in Fig 8, 200 ng/ml IL-6 induced a statistically significant, 40% increase in transepithelial leak of ^14^C-D-mannitol, 48 hrs after IL-6 exposure. A simultaneous incubation of 5, 10 or 50 μM RA with IL-6 completely blocked the IL-6-induced leak to ^14^C-D-mannitol. However, unlike the effect of TNF, a corresponding decrease of TER was not observed as a result of IL-6 exposure.

## Discussion

Retinoic acid produced the following effects on 16HBE cell layers: 1) increase of basal barrier strength evidenced by an 89% increase in TER and 22% decrease in mannitol flux (Fig 1); 2) moderate but significant suppression of endogenous TNF-α production (Fig 2); 3) attenuation of TNF-α-induced barrier leak to mannitol, lactulose and inulin (small and moderately high molecular weight probes)(Figs 3 and 4); 4) attenuation of the IL-6-induced increase in barrier leak to mannitol; 5) preservation of occludin and claudin-4 immunostaining in the presence of TNF-α (Fig 5); 6) reversal of the downward “band-shift” of occludin triggered by TNF-α (Fig 6); and 7) inhibition of the increase in ERK-1,2 phosphorylation induced by TNF-α (Fig 7).

As mentioned above, RA has been shown to be beneficial to barrier function in several other epithelial models both *in vitro* and *in vivo* including oral, blood-retinal, blood-brain, and intestinal barriers [17–20]. To our knowledge, this is the first study showing improvement in airway basal barrier function using RA. Suppression of endogenous TNF-α production may be partially responsible for RA’s induced elevation in basal barrier strength given TNF-α’s detrimental effect on the 16HBE barrier (Figs 1 and 2). While only TNF-α production was assessed here, RA could also be dampening production of other cytokines as is the case in mouse hepatocytes, in human and mouse adipocytes, in rats with collagen-induced arthritis, and in the kidneys of diabetic rats [33–37]. More experimentation would be needed to reveal the distinct mechanisms involved in RA enhancing barrier function of 16HBE cell layers, but the physiological results presented here do suggest that RA could be useful prophylactically in preventing lung barrier dysfunction *in vivo*.

TNF-α has been shown to produce barrier leak in a variety of other different epithelial cell models [6, 26, 38–43]. The purpose of this study—with respect to TNF-α-induced barrier leak—was not to delve into the molecular mechanisms of how TNF-α produces leak in 16HBE cell layers, but rather initially characterize the type of leak that occurs. Transepithelial leak can be produced by either perturbation of TJs or through the creation of actual holes in the epithelial sheet arising from cell death and detachment [3, 38, 39]. Here, we note that barrier compromise triggered by TNF-α correlated with changes in TJ proteins.

While it is unclear whether TNF-α is producing actual holes in 16HBE cell layers, the physiological data collected here may suggest such holes are present. In LLC-PK_1_, renal cell layers, both TNF-α and the phorbol ester, TPA, induced leak [40]. However, the leak produced by these two inducers exhibited very different characteristics [40]. A size limit existed as to which probe molecules manifested increased leak triggered by TNF-α, while no such size limit existed for TPA-induced leak. The presence of holes (or complete loss of TJs) created by TPA would be consistent with the absence of any size limitation, while the lack of holes in TNF-α treated cell layers would result in only low MW probes crossing cell layers [40]. Since no size limit was observed for TNF-α-induced leak in 16HBE (increased leak for mannitol, lactulose and inulin), holes may be present in addition to alterations in TJs. In any event, what is most important is that RA almost completely restores barrier strength of this airway model in the presence of TNF-α to all three probes (Figs 3 and 4). The implications of this could be very important for patients with high systemic levels of cytokines, placing them at an increased risk of lung water accumulation and subsequent respiratory distress. This data suggests that RA could be helpful either therapeutically or prophylactically.

Occludin expression and localization have been shown to be causal to changes in transepithelial permeability, with higher expression of occludin at TJs correlating with decreased permeability/leak [44]. Further, the degree of phosphorylation of occludin correlates with its localization, with increased phosphorylation of occludin associating with TJ localization and less phosphorylation associating with non-TJ localization [45]. Here, immunostaining of occludin was greatly diminished in TNF-α-treated cell layers (Fig 5). Additionally, TNF-α caused a downward “band-shift” of occludin, suggesting a decrease in occludin phosphorylation (Fig 6), though this contrasts with what was observed in hCMEC/D3 cells where an upward-shift of occludin was rapidly initiated by TNF-α [28]. These results could suggest TNF-α-induced leak in 16HBE cell layers is partially carried out through induced change in occludin phosphorylation with subsequent or concurrent loss of occludin from TJs. In addition to altering occludin–and potentially other TJ proteins’—phosphorylation states, TNF-α could also induce paracellular leak by triggering MLCK-dependent endocytosis of TJ proteins [46].

While in this study, occludin is being highlighted and the changes in occludin band densities and junctional staining correlate nicely with physiological results, we by no means suggest that changes in occludin or claudin-4 account for the changes in 16HBE barrier function that we observed here. As we have shown (Figs 1 and 5), TNF- and RA-induced changes in claudin-4 are also pronounced. Investigations into many other TJ proteins are clearly required and will be the subject of future studies by our group. In this regard, it is worth noting that RA is not only reducing the changes in occludin and claudin-4 abundance caused by TNF (Fig 5) but is itself capable of causing increased levels of claudin-4 in control cell layers (Fig 1C). It should be noted however that whereas in immunofluorescence the sharply decreased claudin-4 signal caused by TNF is partially reversed by RA, this was not observed in Western immunoblotting. Therefore, a focal effect regarding RA reversal of TNF action on claudin-4 cannot be ruled out here.

ERK signaling has been shown to influence TJ integrity differently in different epithelia [28, 47]. For example, reducing ERK-1,2 activity in MDCK (canine kidney) and LLC-PK_1_ (porcine kidney) epithelial cell layers promotes TJ assembly, while increased ERK-1,2 activity appears to be protective concerning barrier function in differentiated CACO-2 (human intestinal) epithelial cell layers [48, 49]. Our data indicates that TNF-α increases ERK-1,2 phosphorylation and that RA can inhibit this phosphorylation increase. Future work is needed to show whether these observed changes in ERK-1,2 phosphorylation are in fact causal to the barrier function changes observed here.

In all data reported here, we are showing time points at which observed effects were most obvious. These can be 24 or 48 hrs. or as short as 30 mins in the case of ERK phosphorylation. It is worth noting that we are not always reporting on the same induced phenomena. The mechanisms by which RA improves basal barrier function may not be the same by which RA opposes TNF-induced barrier compromise. They may quite likely have differing time courses. Similarly, the effects of RA on TJ proteins’ expression or phosphorylation may have a different time course than effects on actual barrier function.

In the midst of the current SARS-CoV-2 pandemic, it is worth noting that an increasing published literature suggests a significant source of COVID-19 morbidity may be traceable to a “cytokine storm” that occurs in a meaningful percentage of infected patients [50–53]. Furthermore, evidence for an association between the cytokine storm and resulting lung water may be building [32, 54]. It is therefore very noteworthy that our work indicates that RA may not only be capable of improving basal lung barrier function but also protecting lung barrier function during assault by cytokines such as TNF-α and IL-6. This therefore suggests the potential for RA as a prophylactic and therapeutic adjuvant therapy in COVID-19 treatment, alongside direct anti-viral therapies.

## Conclusion

More work is required to identify molecular mechanisms that are responsible for RA’s action on 16HBE barrier function. Work on additional airway epithelial barrier models is needed to determine if the results reported here might accurately reflect in vivo actions. However, the results contained here do suggest that RA may be effective prophylactically in strengthening airway barriers as well as therapeutically in preventing barrier leak triggered by inflammatory cascades and disease pathogens.

## Supporting information

S1 File Raw immunoblot images. This file contains the raw immunoblot images for Figs 6A and 7A and S1 and S2
(PDF)

S2 File Data used throughout manuscript. This file contains the data used to construct all graphs as well as the effects of retinoic acid on 16HBE barrier function after 24-hours of exposure.
(XLSX)

S1 Fig Effect of 1-hour TNF-α or TNF-α + retinoic acid on phosphorylated ERK-1,2 expression in 16HBE. Confluent cell layers were treated with control or 50μM RA-supplemented medium for 24-hours prior to treatment with TNF-α or TNF-α + RA for 1 hour. Phosphorylated ERK-1,2 immunoblots were prepared and band densities were quantified as described in Materials and Methods. * indicates P < 0.05, **indicates P < 0.01 (Student’s t test, two-tailed).
(TIF)

S2 Fig Effect of 4-hour TNF-α or TNF-α + retinoic acid on phosphorylated ERK-1,2 expression in 16HBE. Confluent cell layers were treated with control or 50μM RA-supplemented medium for 24-hours prior to treatment with TNF-α or TNF-α + RA for 4 hours. Phosphorylated ERK-1,2 immunoblots were prepared and band densities were quantified as described in Materials and Methods.
(TIF)
