# Supplementary material for: Retinoic acid improves baseline barrier function and attenuates TNF-α-induced barrier leak in human bronchial epithelial cell culture model, 16HBE 14o-
Source: PLoS One. 2020 Dec 10;15(12):e0242536. doi: 10.1371/journal.pone.0242536 (PMC7728186; doi:10.1371/journal.pone.0242536)
Supplement: S1 File — This file contains the raw immunoblot images for Figs 6A and 7A and S1 and S2 (PDF) [file pone.0242536.s001.pdf]

Image used to generate Fig 6A:

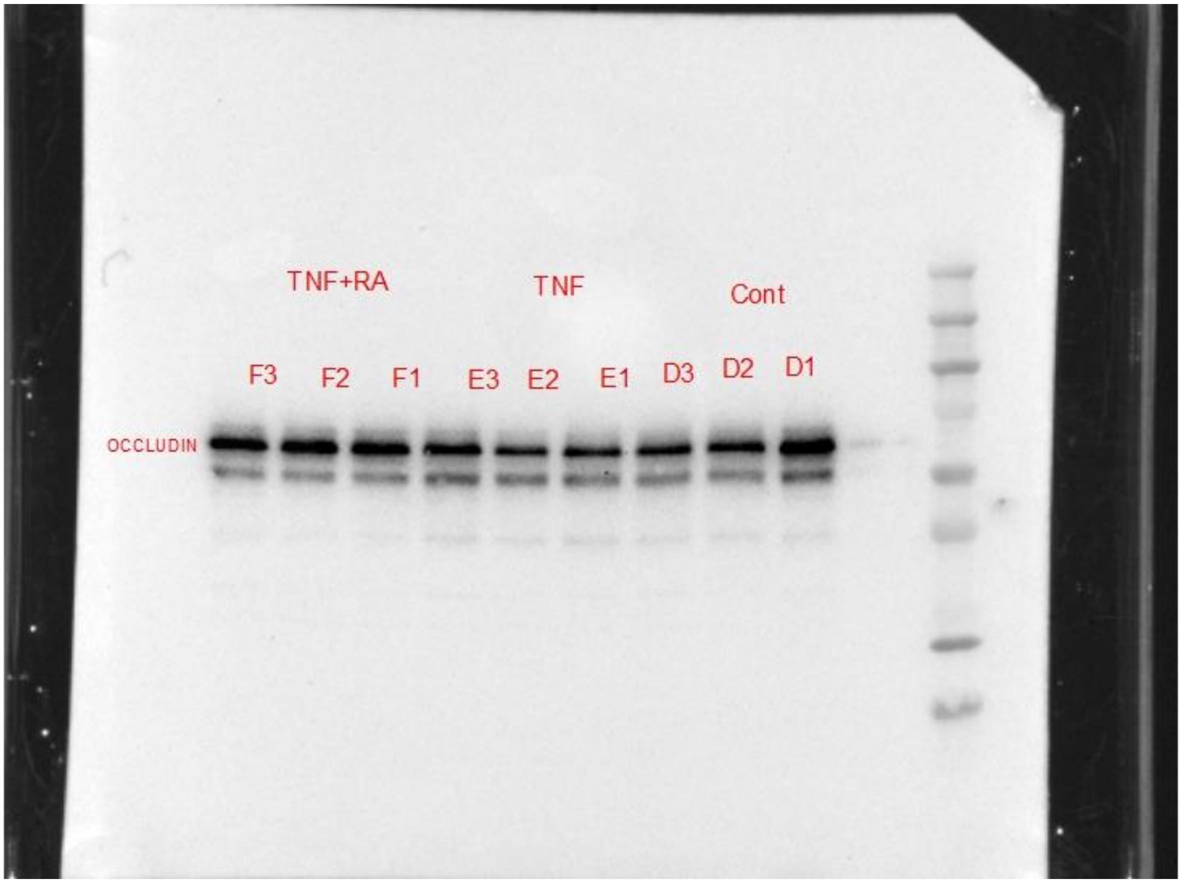

Control cell samples [lanes D1-D3] TNF-α-treated cell samples [lanes E1-E3], and TNF-α + RA-treated cell samples [lanes F1-F3].

Loading order: Right to left.

Method of obtaining image: BioRad ChemiDoc Imaging System

Image used in Fig7A:

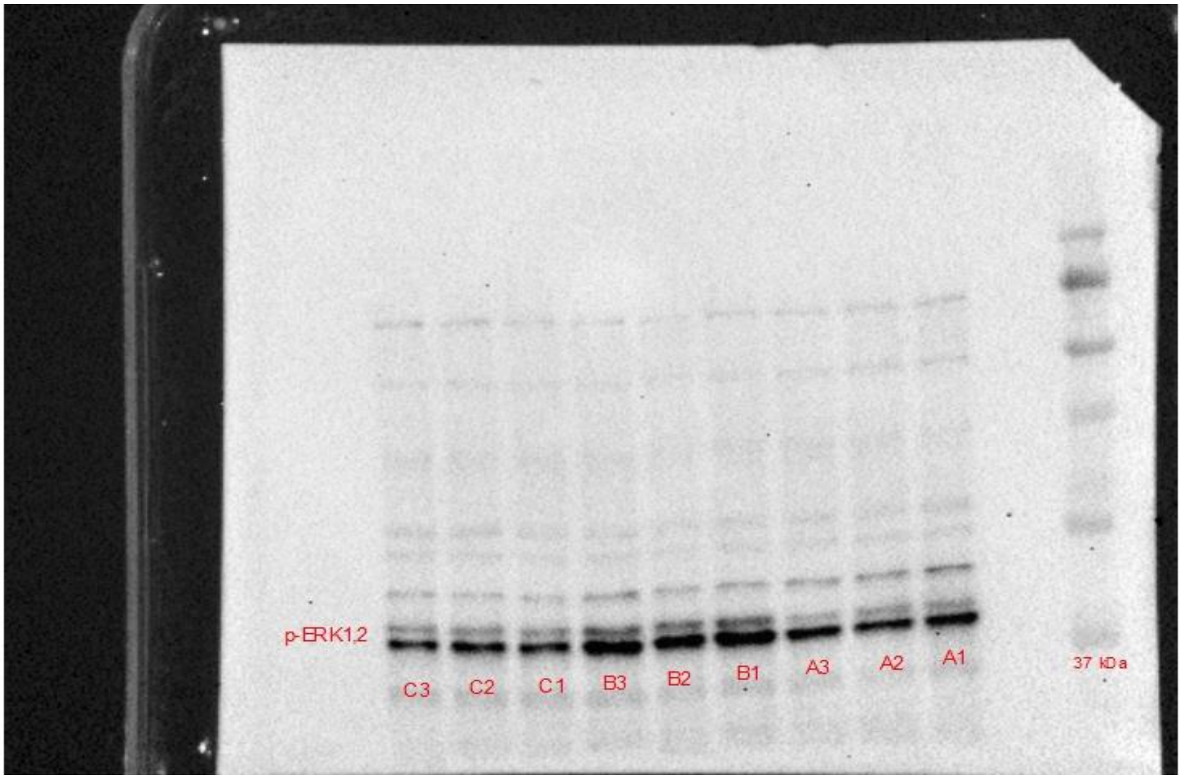

Control cell samples [lanes A1-A3] TNF-α-treated cell samples [lanes B1-B3], and TNF-α + RA-treated cell samples [lanes C1-C3].

Loading order: Right to left.

Method of obtaining image: BioRad ChemiDoc Imaging System

Image used to generate S1 Fig:

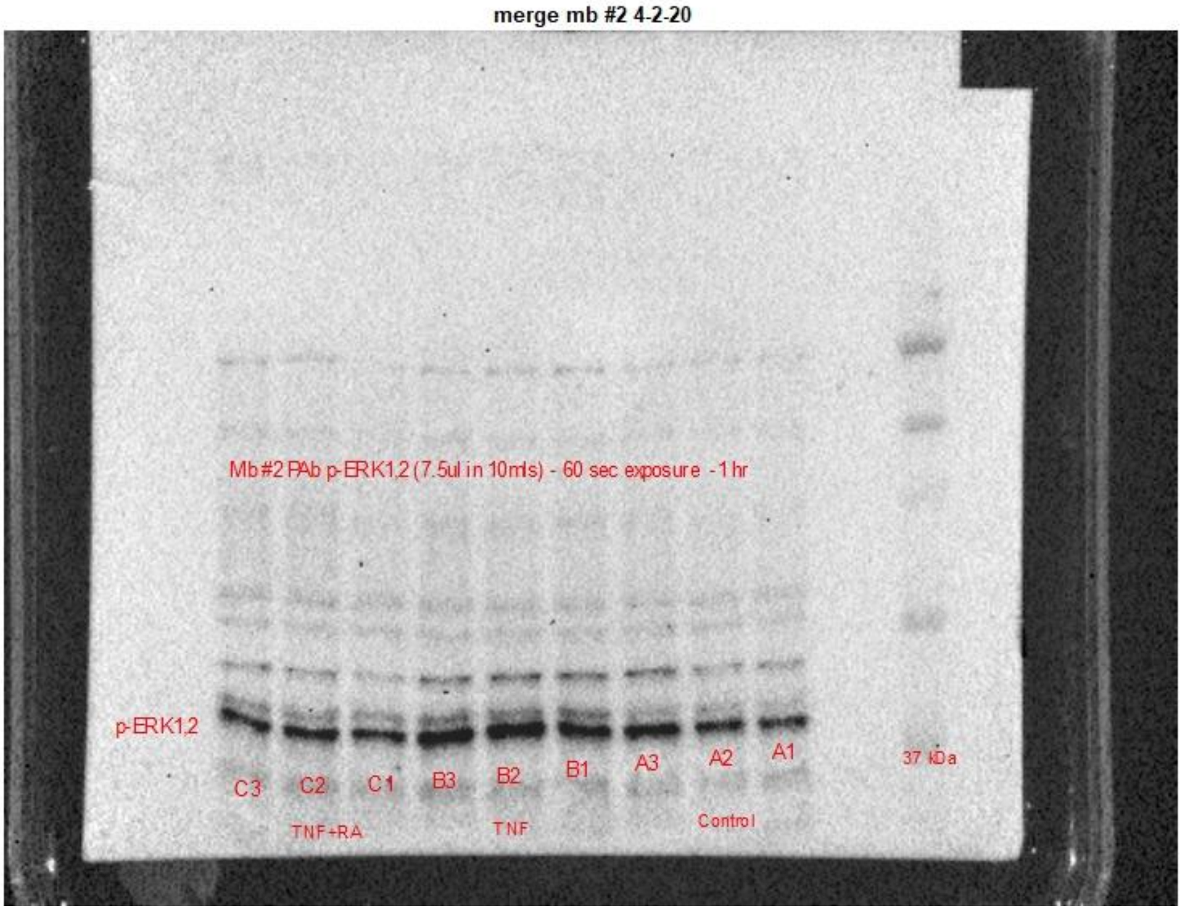

Control cell samples [lanes A1-A3] TNF-α-treated cell samples [lanes B1-B3], and TNF-α + RA-treated cell samples [lanes C1-C3].

Loading order: Right to left.

Method of obtaining image: BioRad ChemiDoc Imaging System

Image used to generate S2 Fig:

merge mb #2 3-2020

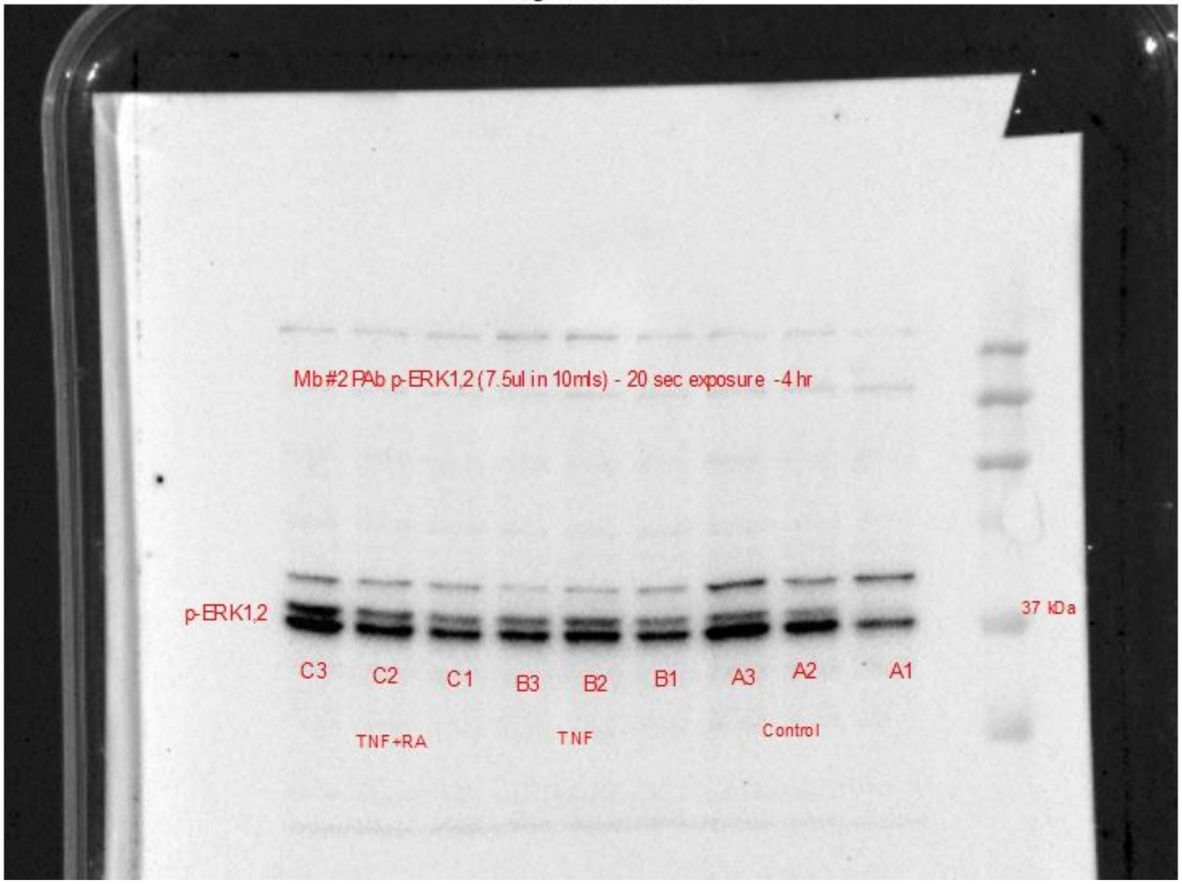

Control cell samples [lanes A1-A3] TNF-α-treated cell samples [lanes B1-B3], and TNF-α + RA-treated cell samples [lanes C1-C3].

Loading order: Right to left.

Method of obtaining image: BioRad ChemiDoc Imaging System
