# Supplementary figures and images for: Retinoic acid improves baseline barrier function and attenuates TNF-α-induced barrier leak in human bronchial epithelial cell culture model, 16HBE 14o-
Source: PLoS One. 2020 Dec 10;15(12):e0242536. doi: 10.1371/journal.pone.0242536 (PMC7728186; doi:10.1371/journal.pone.0242536)

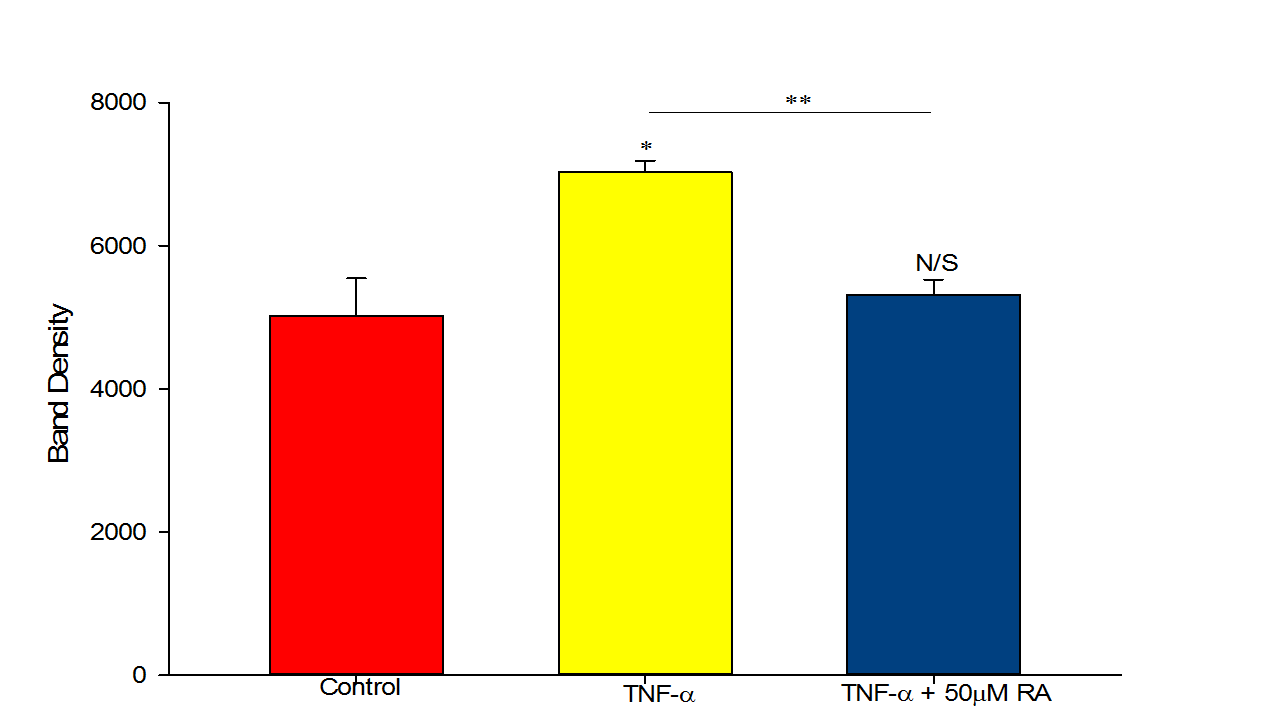

Supplement: S1 Fig — Confluent cell layers were treated with control or 50μM RA-supplemented medium for 24-hours prior to treatment with TNF-α or TNF-α + RA for 1 hour. Phosphorylated ERK-1,2 immunoblots were prepared and band densities were quantified as described in Materials and Methods. * indicates P < 0.05, **indicates P < 0.01 (Student’s t test, two-tailed). (TIF) [file pone.0242536.s003.tif]

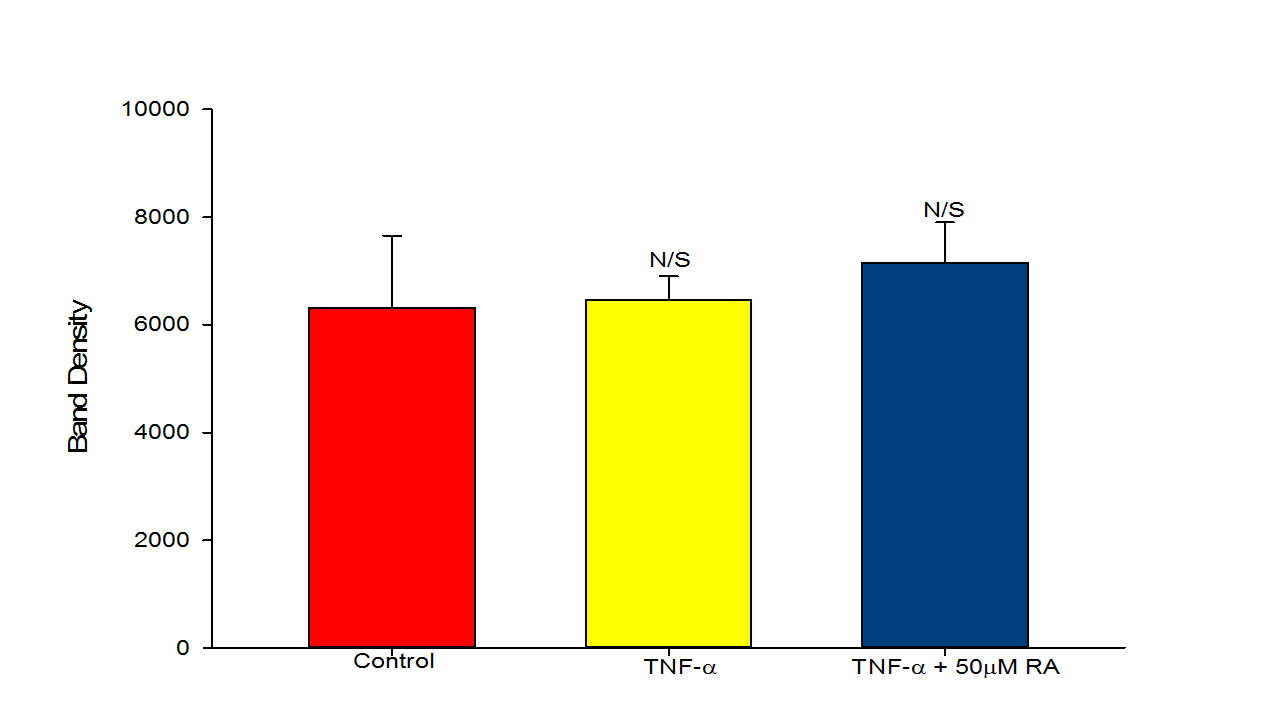

Supplement: S2 Fig — Confluent cell layers were treated with control or 50μM RA-supplemented medium for 24-hours prior to treatment with TNF-α or TNF-α + RA for 4 hours. Phosphorylated ERK-1,2 immunoblots were prepared and band densities were quantified as described in Materials and Methods. (TIF) [file pone.0242536.s004.tif]
